# The Homiletic Review

**Published:** 1888-11

**Authors:** 


THE HOMILETIC REVIEW for November has a masterly critical article on Dr. Maclaren, of Manchester, England, one of the greatest living preachers. It forms the ninth of the remarkable series which has appeared in the *Review* from the same pen. Dr. Schaff gives the first of two papers on Chrysostom, "the greatest preacher of the Greek Church." Dr. Behrends ably discusses "Miracles" in relation to Christian evidences as affected by modern criticism. Dr. Lyman Abbott gives a very sensible article on "The Church of our Workingmen," while Dr. Pierson presents a bright "Cluster of Gems," illustrated of "Truth." There are two grand sermons by Dr. Herrick Jonhson, of Chicago, and Dr. S. E. Herrick, of Boston, the former on "The Influence of the Church of God," and the latter on "The Trial of Christ's Personal Virtue." Among the other six sermons is a very striking one by the Lord Bishop of Meath, reported for the *Review*. The European Department is specially full and interesting this month. The other parts of the number are up to mark. Published by Funk & Wagnalls, 18 and 20 Astor Place, New York. $3 00 per year; 30 cents per single number.

